# Transcriptome Analysis of Gene Expression during Chinese Water Chestnut Storage Organ Formation

**DOI:** 10.1371/journal.pone.0164223

**Published:** 2016-10-07

**Authors:** Libao Cheng, Shuyan Li, Sainan Chen, Yan Wang, Meizhen Yu, Xuehao Chen, Liangjun Li, Jingjing Yin

**Affiliations:** 1 School of Horticulture and Plant Protection of Yangzhou University, Yangzhou, Jiangsu 225009, People’s Republic of China; 2 Guangling College of Yangzhou University, Yangzhou, Jiangsu 225009, People’s Republic of China; Kunming University of Science and Technology, CHINA

## Abstract

The product organ (storage organ; corm) of the Chinese water chestnut has become a very popular food in Asian countries because of its unique nutritional value. Corm formation is a complex biological process, and extensive whole genome analysis of transcripts during corm development has not been carried out. In this study, four corm libraries at different developmental stages were constructed, and gene expression was identified using a high-throughput tag sequencing technique. Approximately 4.9 million tags were sequenced, and 4,371,386, 4,372,602, 4,782,494, and 5,276,540 clean tags, including 119,676, 110,701, 100,089, and 101,239 distinct tags, respectively, were obtained after removal of low-quality tags from each library. More than 39% of the distinct tags were unambiguous and could be mapped to reference genes, while 40% were unambiguous tag-mapped genes. After mapping their functions in existing databases, a total of 11,592, 10,949, 10,585, and 7,111 genes were annotated from the B1, B2, B3, and B4 libraries, respectively. Analysis of the differentially expressed genes (DEGs) in B1/B2, B2/B3, and B3/B4 libraries showed that most of the DEGs at the B1/B2 stages were involved in carbohydrate and hormone metabolism, while the majority of DEGs were involved in energy metabolism and carbohydrate metabolism at the B2/B3 and B3/B4 stages. All of the upregulated transcription factors and 9 important genes related to product organ formation in the above four stages were also identified. The expression changes of nine of the identified DEGs were validated using a quantitative PCR approach. This study provides a comprehensive understanding of gene expression during corm formation in the Chinese water chestnut.

## Introduction

The Chinese water chestnut belongs to the sedge family, and is widely cultivated throughout the world, including Asia, Australia, Africa, and the Pacific and Indian oceans. It has many uses, such as water purification and embankment protection, and its corms are also edible [1,2,3,4]. Indeed, the water chestnut is a popular aquatic vegetable because of its unique taste and the fact that it is fat-, gluten-, and cholesterol-free. It is also a critical ingredient in traditional Chinese medicine [5].

Similar to other aquatic species, this plant must be maintained in pools, water gardens, tanks, or shallow water tubs in greenhouses during the entire growth season from spring to autumn. It is often asexually propagated by directly planting a corm with a healthy apical bud into wet soil. The corm not only provides nutrition but also serves as an energy unit during the early growth of the plant. In its early developmental stage, buds first form at the end of the main stem, and then develop into stolons. The corms form at the tips of each stolon [6].

The genetic and morphometric processes involved in the formation of storage organs have been extensively studied over past decades [7,8]. Chinese water chestnuts and arrowheads share a similar developmental process in terms of underground stem morphology [9], and corm formation in both species includes an induction stage, an initial swelling stage, and a swelling stage. In the induction stage, rapid radial growth of the stolon tips occurs. In the initial stage, elongation of the stolon is terminated and the tip of the stolon begins to swell. In the swelling stage, the volume of the corm increases and nutrients such as polysaccharides, fats, and proteins are synthesized and deposited [10].

Underground formation of the storage organ can be affected by various internal and environmental factors [11] such as the photoperiod. Storage organ formation of the arrowhead is clearly regulated by the photoperiod, because short days accelerate the process of storage organ formation, whereas long days inhibit it [12]. Perceiving and responding to the photoperiod is the first step for the plant to adjust its growth and developmental processes. The leaf is the main organ that perceives the photoperiod, and the signal is then transferred to the shoot apex through the phloem. The storage organ forms at the tip of the stolon during short days. Phytochrome B (PHYB) is thought to be responsible for photoperiodic responses, and participates in storage organ development during short days. Downregulated PHYB expression leads to the promotion of storage organ formation during short or long days after elimination of the inhibition on tuberization originating from the effect of long days [13]. Flowering locus T (FT), CONSTANS (CO), and GIGANTEA are also involved in photoperiodic regulation during storage organ formation, and the CO/FT module has been shown to be required for the photoperiodic control of tuberization [14,15,16].

The photoperiod shows a relationship with gibberellic acid (GA) to control storage organ formation. Transgenic plants carrying the GA oxidase gene promote underground formation of the storage organ only under conditions of a short-day photoperiod, while earlier formation of storage organs occurs when this enzyme activity is inhibited [17]. During short days, a decrease in the gibberellin content by transgenic StBEL5 and KNOX leads to earlier formation of storage organs [18]. As well as GA, some phytohormones such as cytokinin, jasmonic acid (JA), abscisic acid, indole acetic acid, and ethylene have been identified as being involved in the formation of product organs [19,20,21,22]. For instance, axillary buds decapitated from peas and potatoes were inhibited by the exogenous application of auxin [23]. Ethylene is involved in many plant developmental processes [24,25], including the induction of storage organ formation and root bulking in carrots [26]. JA has also been shown to play an important role in plant biological processes [27]. Several studies have shown that JA has a positive effect on the swelling of underground storage organs. Endogenous JA is believed to be a strong inducer of storage organ development in dicotyledonous potato plants and monocotyledonous yam plants [28]. Moreover, Ravnikar et al. (1993) showed that exogenous JA functions in the formation of garlic bulbs [29].

Despite its importance, particularly in south China, few studies have been carried out to investigate underground corm growth of the Chinese water chestnut. Formation of the storage organ is genetically regulated, but the expression of genes involved in corm formation has not been studied in detail, although work in other species has partially described the above processes, and many storage organ-related genes have been documented [30]. An understanding of the genes expressed during corm formation is necessary to provide insights into the mechanism of corm development, which may lead to better regulation of production in the future.

DNA sequencing is an efficient approach to gain important information on genes, genetic variation, and gene function for biological processes [31,32]. Recently, many critical genes regulating growth and metabolism have been identified using a tag sequencing technique in horticultural plants [33,34]. In the present study, gene expression analysis at the transcriptional level was performed to monitor changes in biological processes during the underground formation of the storage organ [10]. Differentially expressed genes (DEGs) from four developmental stages of the Chinese water chestnut were sequenced and analyzed, with the aim of comprehensively understanding the process of corm formation.

## Materials and Methods

### Plant Materials

‘Hongbaoshi’, a species of Chinese water chestnut that is widely cultivated in China, was planted in fields at a water depth of 5–10 cm in spring, with average temperatures of 30°C in the day and 20°C at night during the entire growth season. Four or more stolons were observed to develop and elongate in the correct order on each plant. About 90–100 days after plantation, corm formation started at the stolon tips. For tag sequencing and gene expression analysis, corms from the four developmental stages (stolon, initial swelling, middle swelling, and later swelling stage) (S1 Fig) were used (four tips from different plants were combined for each stage). To obtain materials from different developmental stages, water chestnuts were cultivated in a field (non-private) located in southeastern China. Permission for sample collection was granted from the Department of Horticulture of YangZhou University, China. No specific permissions were required for the location or the field studies because the experiments did not involve any endangered or protected species.

### Screening Differentially Expressed Genes

Corm transcriptomes from the four developmental stages were analyzed. Stolon tips and corms from the initial swelling, middle swelling, and later swelling stages were collected and ground, and RNA was isolated using an RNA extraction mini kit (Qiagen, Hilden, Germany). DNase I was added to eliminate DNA contamination. Sequencing of transcripts in the form of special constructs was completed by the Beijing Institute of Genomics.

To screen the DEGs, transcriptomes from the four stages were analyzed with the aim of tracking major changes in metabolism. DEG libraries of the four samples were determined in parallel using Illumina gene expression sample preparation kits. Briefly, total RNA of these four stages was used for mRNA capture with magnetic oligo(dT) beads. First and second strand cDNA were synthesized, and the bead-bound cDNA was subsequently digested with *Nla*III.

3′-cDNA fragments attached to oligo(dT) beads were ligated to Illumina GEX NlaIII adapter 1, which contained a recognition site for the endonuclease *Mme*I to enable cleavage 17 bp downstream of the recognition site (CATG) to produce tags with adapter 1. After removing the 3′ fragment with magnetic bead precipitation, an Illumina GEX adapter 2 was introduced at the *Mme*I cleavage site. The resulting adapter-ligated cDNA tags were amplified using PCR primers that were annealed to the adaptor ends for 15 cycles.

The 90 bp fragments were sequenced separately at the 3′ and 5′ ends of the gene (PE90), and the PCR product was recovered and purified from a 6% polyacrylamide Tris-Borate-EDTA gel. The final quality of the tagged sequences was checked using an Agilent 2100 Bioanalyzer. The four tag libraries constructed underwent Illumina proprietary sequencing for cluster generation through *in situ* amplification, and were deep sequenced using an Illumina Genome Analyzer. For the raw data, we filtered out adaptor sequences, low-quality tags (tags with unknown nucleotides N), empty reads, tags that were too short or too long, and tags with only one copy, to obtain clean tags. The types of clean tags were represented as distinct clean tags. Subsequently, we classified the clean tags and distinct clean tags according to their library copy number, determined their percentage in the total clean and distinct tags, and analyzed the saturation of the four libraries.

Transcriptome *de novo* assembly was carried out with a short read assembling program, Trinity [35]. This is comprised of three independent software modules including inchworm, chrysalis, and butterfly. RNA-seq data were assembled into unique sequences of transcripts for a dominant isoform, then these sequences were partitioned into many individual De Bruijn graphs (each graph represented the transcriptional complexity at a given gene or locus). Unigenes were obtained after each graph was independently processed to extract full-length splicing isoforms and to tease apart transcripts derived from paralogous genes. For annotation, all tags were mapped to the reference sequence from the NCBI database (http://www.ncbi.nlm.nih.gov/), and no more than 1 bp of nucleotide mismatch was allowed. The alignment procedures were conducted by following the protocols described in the online documentation (http://maq.sourceforge.net) and adopting default parameters. To monitor mapping events on both strands, sense and complementary antisense sequences were included in the mapping process. The tags mapped to reference sequences from multiple genes were filtered. The data sets supporting the results of this article are available in the NCBI database, Submission ID: SUB1471108 and BioProject ID: PRJNA318689, which is available at: http://www.ncbi.nlm.nih.gov/bioproject/318689.

### Identification of Differentially Expressed Genes

The transcriptome of the Chinese water chestnut from the four developmental stages was used as a reference for DEG screening and analysis because of the unavailability of existing data. All expressed genes were monitored, and their gene functions were explored using database annotations such as nr, Swiss-Prot, KEGG, and COG, using the criteria of BLASTx alignment (E value < 0.00001) between unigenes and protein databases such as Swiss-Prot, KEGG, and COG. The best aligned results were used to decide the sequence direction and functions of the unigenes.

In case of any conflict between the results from different databases, a priority order of nr, Swiss-Prot, KEGG, and COG was followed when deciding the sequence direction of unigenes. When a unigene aligned with none of the above databases, ESTscan was used to predict its coding region as well as to decide its sequence direction. All of the expressed unigenes were classified according to their functions in metabolic processes. For DEG screening, a false discovery rate (FDR) ≤ 0.001 and the absolute value of the log_2_ ratio ≥ 1 were used as thresholds to judge the significance of the difference in expression of the unigenes. All DEGs were deposited into the NCBI database under GenBank accessions KX365752 to KX365849.

### Gene Expression Analysis by Quantitative PCR

To study gene expression, the cultivation conditions of the Chinese water chestnut were maintained as described above. Quantitative (q)PCR analysis was performed to quantify the transcriptional level of nine novel genes of the Chinese water chestnut at the stolon stage, initial stage, middle swelling stage, and later swelling stage, to evaluate the results of tag sequencing. Total RNA was extracted from 100 mg samples of stolon tips and corms at the initial swelling, middle swelling, and later swelling stages using an RNA extraction mini kit (Qiagen). Approximately 10 samples at each stage were the pooled. DNase I was used to digest DNA during the RNA extraction process to eliminate DNA contamination. A total of 1–2 μg of RNA was used for cDNA synthesis according to the manufacturer’s instructions (Promega, Madison, WI). qPCR was performed with the Mx3000P machine (American, agilent). SYBR Green Master Mix was used to identify mRNA levels according to the manufacturer’s instructions (Tiangen, Beijing, China). According to the sequencing results, primers were designed for genes that showed enhanced transcription during corm formation (S1 Table). β-Actin was used as an internal standard and amplified using the following primers: forward 5′-AACCTCCTCCTCATCGTACT-3′, and reverse 5′-GACAGCATCAGCCATGTTCA-3′. Amplification was performed in a 25 μL reaction mixture, containing 12.5 μL SYBR *Premix Ex Taq* II (Tli RNaseH Plus) (2×), 10 μM of each of the forward and reverse primer, 2 μL RT reaction solution (cDNA), and 8.5 μL dH_2_O. The PCR program consisted of 94°C for 30 s, then 40 cycles of 95°C for 5 s and 60°C for 60 s. qPCR reactions were performed in triplicate.

## Results

### Transcriptome Profile during Corm Formation

**Fig 1:**
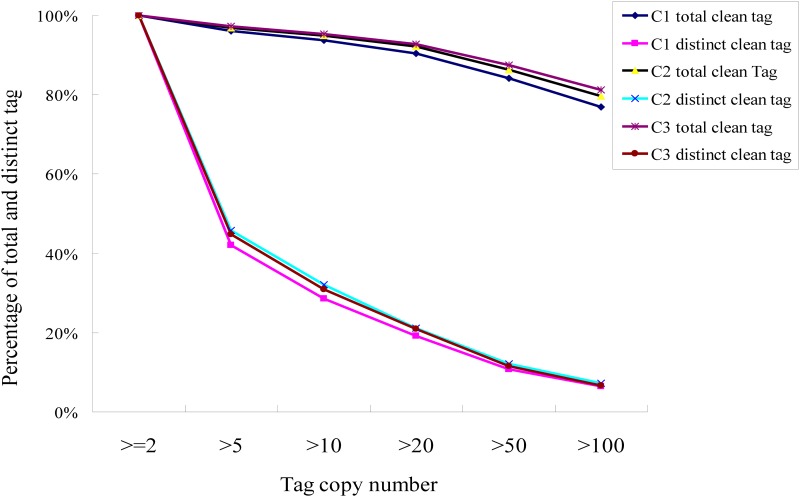
Distribution of the total clean tags and the distinct clean tags from the four libraries.

Four libraries were constructed from the four stages of corm development (the stolon stage, the initial swelling stage, the middle swelling stage, and the swelling stage) using the Illumina sequencing platform, with the aim of identifying genes relevant to corm development in the Chinese water chestnut. The transcriptomes of the four developmental stages of the corm were preprocessed before mapping the tags of each stage. About five million tags were obtained in the B1, B2, B3, and B4 libraries, with 283,469, 245,690, 209,624, and 251,126 distinct tags, respectively. Additionally, 4,371,386, 4,372,602, 4,782,494, and 5,276,540 clean tags were obtained, including 119,676, 110,701, 100,089, and 101,239 distinct clean tags in the B1, B2, B3, and B4 libraries, respectively, after filtering all raw tags with reference sequences. A detailed analysis of the tags in each library is shown in Table 1. We found that the copy distribution of the total and distinct clean tags in the four libraries was similar, with about 5% of distinct clean tags at higher than 100 copies, and about 30% of tags at 5–50 copies. The number of distinct clean tags at between two and five copies (about 60%) was higher than that of other libraries (Fig 1). To evaluate whether the sequenced tags were sufficient to cover the whole transcriptome, analysis of sequencing saturation was also carried out for the four libraries of the different developmental stages. We observed that the number of detected genes increased until the sequencing tags reached approximately four million (S2 Fig), suggesting that the tags identified in each library were sufficient to reflect the entire transcriptional information of the Chinese water chestnut genome.

**Table 1:** Categorization and abundance of tags.

		B1	B2	B3	B4
Raw Tag	Total number	4728376	4716581	4969688	4427680
	Distinct Tag	283469	245690	209624	251126
Clean Tag	Total number	4371386	4372602	4782494	5276540
	Distinct Tag number	119676	110701	100089	101239
All Tag Mapping to Gene	Total number	3147933	3200198	3905586	4571911
	Total percentage of clean tag	72.01%	73.19%	81.66%	86.65%
	Distinct Tag number	59110	55905	53726	59622
	Distinct Tag percentage of clean tag	49.39%	50.50%	53.68%	57.81%
Unambiguous Tag Mapping to Gene	Total number	2348643	2114294	1926958	795588
	Total percentage of clean tag	53.73%	48.35%	40.29%	45.08%
	Distinct Tag number	47086	44427	42461	46419
	Distinct Tag percentage of clean tag	39.34%	40.13%	42.42%	45.71%
All Tag-mapped Genes	Total number	20222	19877	19518	15486
	Percentage of reference genes	52.93%	52.02%	51.08%	40.53%
Unambiguous Tag-mapped Genes	Total number	14920	14581	14260	10706
	Percentage of reference genes	39.05%	38.16%	37.32%	28.02%
Unknown Tag	Total number	1223453	1172404	876908	704629
	Total percentage of clean tag	27.99%	26.81%	18.34%	13.35%
	Distinct Tag number	60566	54796	46363	41617
	Distinct Tag percentage of clean tag	50.61%	49.50%	46.32%	42.19%

In total, 38,207 unigenes with an average length of 788 bp were obtained following assembly of these clean tags. Gene functions were identified using BLASTx software by comparison with the existing NCBI database, using a cut-off E value of 10–5. Of these, about 40% of all distinct sequences in the four libraries showed an above cut-off BLAST result, and about 60% did not match with known genes. All matched genes were grouped into 26 catalogues according to their annotated functions. We found that the function of catalogue with the largest number of genes was predicted, and the group with the smallest number of genes was relevant to extracellular structure (S3 Fig). The DEGs were also classified into three categories according to their function: biological process, cellular component, and molecular function. We found that the DEGs (from stolon stage to late swelling stage) were involved in various metabolisms. The number of genes in the biological process category related to cellular process and metabolism process was greater than for other processes. It was also shown that most genes in the cellular component were involved in cell, cell junction, organelle, organelle part, and membrane functions. For molecular function, many genes participated in binding, catalytic activity, and transport activity (S4 Fig).

### Differentially Expressed Genes in the Four Stages of Corm Formation

#### Changes in gene expression during corm formation

**Fig 2:**
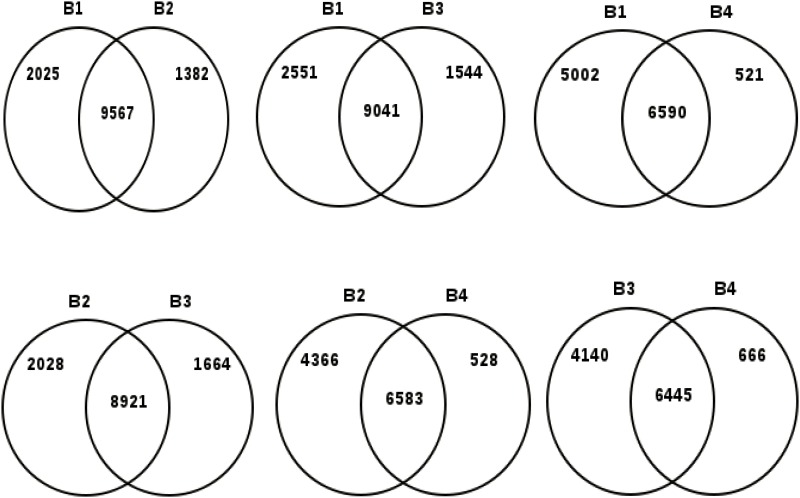
Analysis of tag-mapped genes of the four corm developmental stages in the Chinese water chestnut.

To monitor the changes in gene expression, four libraries at different developmental stages of the corm were constructed. A total of 11,592, 10,949, 10,585, and 7,111 transcripts were identified from the B1, B2, B3, and B4 libraries, respectively. Of these, 9,567, 8,921, and 6,445 genes were similarly expressed in the B1/B2, B2/B3, and B3/B4 stages, respectively (Fig 2 and S2 Table). Within these four libraries, we also found a large number of genes that changed expression during corm development, suggesting that the storage organ formation process is highly complex at the molecular level. The number of genes per million clean tags was used to evaluate the abundance of transcripts in the four libraries.

In the B1/B2 stages, 1,027 genes changed expression; of these, the expression of 315 genes was enhanced and that of 712 genes was decreased. In the B2/B3 stages, 182 genes were identified as upregulated and 1,014 were downregulated. In the B3/B4 stages, we observed enhanced expression of 106 genes and decreased expression of 3,213 genes. In summary, the number of transcripts in the B1/B2 libraries showed a smaller change compared with the B1/B4 libraries, indicating that the development of the corm from the stolon stage to the initial swelling stage differs from that from the initial swelling stage to the swelling stage (Table 2).

**Table 2:** DEGs across all libraries. All genes mapped to the reference sequence and genome sequences were examined for their expression differences across different libraries. The numbers of differentially expressed genes represent transcripts, using threshold values of FDR ≤ 0.001 and log2 ratio ≥ 1 for controlling false discovery rates. B1, B2, B3, and B4 represent the samples that were collected at the stolon stage, the initial swelling stage, the middle swelling stage, and the later swelling stage of the corm, respectively.

	B1:B2	B1:B3	B1:B4	B2:B3	B2:B4	B3:B4
Total	1027	2294	4933	1196	4099	3319
Up-regulated	315	417	145	182	61	106
Down-regulated	712	1877	4788	1014	4038	3213

#### Differentially expressed genes at each stage

During corm development of the Chinese water chestnut, we found that a large number of genes altered their expression at the B1/B2, B2/B3, and B3/B4 stages. Therefore, 20 DEGs showing high levels of change were selected at the B1/B2, B2/B3, and B3/B4 stages to monitor the metabolic process during corm formation.

We observed that two types of hormone, indole acetic acid (indole-3-acetic acid-amido synthetase) and gibberellin (gibberellin 20-oxidase), were involved in the process from the stolon stage to the initial formation stage. A cation/calcium exchanger showed enhanced expression during this period, suggesting that Ca^2+^ has a close relationship with corm formation. We also found increased expression of malic enzyme participating in energy metabolism. In the B2/B3 libraries, two genes were involved in storage metabolism: the glutathione s-transferase gene and the soluble starch synthase gene. We also found altered expression of extensin protein, which is related to corm swelling. Some genes such as calmodulin-like protein and calcium-dependent protein kinase involved in Ca^2+^ signal regulation demonstrated increased transcriptional levels from the initial swelling stage to the middle swelling stage. In the B3/B4 libraries, we found that four genes showed enhanced expression, glutathione s-transferase, starch branching enzyme, aldose reductase-related protein, and proline-rich receptor-like protein kinase, which are involved in substance metabolism. Two genes, ATPase and NADP-dependent malic enzyme, relevant to energy metabolism also showed increased transcriptional levels (Table 3).

**Table 3:** The 20 most differentially expressed annotated genes in the B1/B2, B2/B3, and B3/B4 libraries, based on expressed tag frequency.

Gene ID	Abundant (TMP ratio)	P-Value	FDR	Function annotation
In B1/B2 stages				
B4958	10.13	0	0	Putative zinc finger protein
B512	9.84	0	0	Glycine-rich Cell wall protein
B8571	9.10	0	0	Cytochrome P450 monooxygenase CYP706A12
B4367	7.10	0	0	Cation/Calcium exchanger 3-like
B9242	6.51	0	0	Malic enzyme
B1629	8.21	0	0	Pathogenesis-related protein 10a
B9067	8.10	0	0	Carotenoid cleavage dioxygenase 4, Chloroplastic-like3
B5444	8.10	0	0	Peroxidase 72 precursor
B16992	8.0	0	0.00098	Protein VAC14 homolog
B3517	7.98	0.000018	0.00071	Granule-bound starch synthase
B7629	7.84	0.0012	0.00118	indole-3-acetic acid-amido synthetase
B5325	7.84	0.000057	0.00447	Auxin efflux carrier component 4-like
B4260	7.67	0.000089	0.00124	Plant synaptotagmin
B9579	7.52	0.0088	0.00168	Bzip Transcription factor ABI5
B3968	7.32	0.0041	0.00478	Nodule protein Dg93-like protein
B15737	7.10	0.00058	0.00065	Gibberellin 20 oxidase
B1046	7.10	0.000007	0.0078	Jacalin-like lectin domain containing protein
B9809	7.09	0.000078	0.00045	Exportin-T-like
B4439	7.09	0.000787	0.0089	ATP phosphoribosyl transferase-like
B13033	7.00	0.00897	0.00513	Helicase
In B2/B3 stages				
B6413	11.23	0	0	BTB/POZ and MATH domain-containing protein 2-like
B3872	11.12	0	0	Protein cornichon homolog 1-like
B7997	10.9	0	0	RNA polymerase beta'' subunit, partial
B1399	10.88	0	0	Pathogenesis-related transcriptional activator PTI6
B5860	10.58	0	0	F-box/kelch-repeat protein At5g42350-like
B1587	10.34	0	0	Two-component response regulator ARR9-like
B2484	10.02	0	0	Calcium dependent protein kinase
B3579	9.85	0	00.0008	Extensin
B12950	9.80	0.00072	0	Auxin-responsive family protein
B9404	9.75	0.00007	0.00007	Transcriptional corepressor LEUNIG-like
B2331	9.56	0.000071	0.00005	E3 ubiquitin protein ligase RIE1-like
B13803	9.10	0.000001	0.00004	Cyclin-dependent kinase inhibitor 1-like
B18064	8.54	0.000487	0.00001	Calmodulin-like protein
B18107	7.06	0.00054	0.00047	Soluble starch synthase
B5513	7.05	0.000824	0.00471	NAC protein 1
B15529	6.89	0.000004	0.00015	Malic acid transport protein
B6456	6.65	0.002528	0.00987	NA-directed RNA polymerase II 135 kDa polypeptid
B1561	5.89	0.000006	0.00001	Aldo-keto reductase family 1, member B1
B3517	5.68	0.000212	0.00156	Granule-bound starch synthase
B17903	5.28	0.00009	0.00004	Cellulose synthase BoclesA1
In B3/B4 stages				
B6425	12.58	0	0	Multiprotein-bridging factor
B11692	12.41	0	0	Altered inheritance rate of mitochondria protein 25-like
B5552	11.87	0	0	Cytochrome P450
B15917	11.25	0	0	Heat shock protein
B9585	10.69	0	0	ATPase
B12136	10.58	0	0	Aldose reductase-related protein
B4846	10.25	0	0	Metallothionein-like
B13146	10.02	0	0	Alanine—tRNA ligase
B18689	9.98	0	0.0047	NADP-dependent alkenal double bond reductase P2
B15946	9.87	0.000007	0.00002	Anthocyanidin 5,3-O-glucosyltransferase-like
B6311	9.48	0.000878	0.00047	Glutathione S-transferase
B3976	9.25	0.000045	0.00041	Starch branching enzyme
B11531	8.45	0.000001	0.00787	Mitotic checkpoint serine/threonine-protein kinase
B8401	8.01	0.000147	0.00001	Proline-rich receptor-like protein kinase
B16616	7.88	0.00074	0.00065	NADP-dependent malic enzyme
B16477	7.58	0.00064	0.00001	Shaggy-related protein kinase
B4000	7.14	0.000077	0.00004	Cytochrome P450 CYP84A33
B8396	6.25	0.000877	0.00047	Transcriptional regulator ATRX-like
B7345	6.24	0.000044	0.00001	ABA response element binding factor
B6184	5.21	0.000245	0.00071	Ferritin

We also identified the expression of all transcription factors during corm formation. We found that 15, 16, and eight transcription factors were upregulated in the B1/B2, B2/B3, and B3/B4 stages, respectively (Table 4). In the B1/B2 stages, two of the transcription factors were ethylene-responsive factors (ethylene-responsive transcription factor 9 and AP2 transcription factor), which indicated that ethylene plays an important role from the stolon stage to the initial swelling stage. The expression of some important transcription factors, such as bZIP transcription factor ABI5, WRKY, MYB, and MYC was also found to increase during this period. In the B2/B3 stages, aside from ethylene-responsive factors (ethylene-responsive transcription factor CRF4, AP2 domain class transcription factor, and ethylene-responsive transcription factor 1A), we observed that the expression of CaM-binding transcription factors was also increased. The extensin gene, related to corm swelling, increased its transcriptional level during the initial swelling stage to the middle swelling stage. In the B3/B4 stages, three transcription factors (ERF1 transcription factor, ethylene-responsive transcription factor 5-like, and ethylene-responsive transcription factor 4), involved in ethylene signal transduction, were increased. Additionally, bZIP transcription factor ABI5, transcription factor HBP-1a(c14), and heat shock transcription factor A2 showed enhanced expression during the middle swelling stage to the swelling stage. These gene expression data show that the ethylene signal transduction pathway plays a critical role throughout the entire period of corm formation.

**Table 4:** Expression abundance of some corm formation-related genes identified previously. TMP, transcripts per million clean tags.

Gene ID	P-Value	Function annotation (species)
**B1/B2 libraries**		
B9579	0.00391	bZIP transcription factor ABI5 [Zea mays]
B7116	0.00625	GATA transcription factor [Ricinus communis]
B8501	0.00761	MYB transcription factor MYB17 [Saccharum]
B166	0.0178	WRKY protein [Cucumis sativus]
B13196	0.01537	EIL transcription factor [Zea mays]
B8590	0.0539	Transcription factor ATR2-like [Glycine max]
B13714	0.01250	PHD finger transcription facto [Zea mays]
B4287	0.05680	Zinc finger CCCH domain-containing protein 9 [Brachypodium distachyon]
B9031	0.6587	Trihelix transcription factor GT-2-like [Glycine max]
B2726	0.00	NAC domain protein, IPR003441 [Populus trichocarpa]
B12350	0.0250	MIXTA-like transcription factor [Lotus japonicus]
B16223	0.02500	Heat shock transcription factor [Cenchrus americanus]
B1203	0.0280	AP2 transcription factor [Triticum aestivum]
B490	0.000902	Ethylene-responsive transcription factor 9 [Glycine max]
B16370	0.02670	Transcription factor MYC2-like [Vitis vinifera]
**B2/B3 libraries**		
B13987	0.04060	Trihelix transcription factor GT-1 isoform 1 [Vitis vinifera]
B3355	0.07780	Ethylene-responsive transcription factor CRF4 [Brachypodium distachyon]
B413	0.07780	Scarecrow-like transcription factor PAT1-like [Vitis vinifera]
B6370	0.01480	Homeobox protein HD1 [Zea mays]
B8911	0.01480	Myb-related protein 306 [Brachypodium distachyon]
B8517	0.01480	Transcription factor HBP-1b [Brachypodium distachyon]
B16129	0.0284	GRAS family transcription factor [Populus trichocarpa]
B7244	0.0274	Transcription factor PIF5-like [Glycine max]
B2141	0.0547	GATA transcription factor 26-like [Glycine max]
B2733	0.285	MADS box transcription factor [Elaeis guineensis]
B7071	0.0036	AP2 domain class transcription factor [Malus x domestica]
B40	0.00487	CaM-binding transcription factor [Oryza sativa]
B5637	0.0006	Transcription factor PIF5-like [Vitis vinifera]
B166	0.0128061	WRKY protein [Cucumis sativus]
B3407	0.039235	Ethylene-responsive transcription factor 1A [Medicago truncatula]
B1381	0.0035191	General transcription factor IIH subunit 2-like [Brachypodium distachyon]
**B3/B4 libraries**		
B6147	1.81E-05	Heat shock transcription factor A2 [Vitis vinifera]
B7672	0.011465	ERF1 transcription factor [Vitis pseudoreticulata]
B7672	0.041667	Ethylene-responsive transcription factor 5-like [Vitis vinifera]
B8590	0.1389	Transcription factor BHLH6 [Arabidopsis thaliana]
B9579	0.0156	bZIP transcription factor ABI5 [Zea mays]
B13684	0.0151426	Transcription factor HBP-1a(c14) [Triticum aestivum]
B8331	0.0288674	Transcription factor KAN4-like [Vitis vinifera]
B5811	0.04609146	Ethylene-responsive transcription factor 4 [Zea mays]

#### Expression profiling of genes related to corm formation

RNA sequencing data were compared with previous reports to identify whether our expression model had coverage of well-defined genes. Expression profiling showed that 10 genes were related to corm formation. Detailed expression data of these genes and the biological processes involved in plant metabolism are listed in Table 5. We found that five genes, GIGANTEA, FRUITFUL-like protein, Cycling Dof Factor, BEL1-like HD transcription factor, and Lipoxygenase, showed increased expression during corm swelling, while five were downregulated: zinc finger CONSTANS-like protein, MADS-box transcription factor, SFT family, sucrose synthase, and phytochrome B.

**Table 5:** Expression abundance of some corm formation-related genes identified previously. TMP, transcripts per million clean tags.

Gene ID	Assetion	TPM-B1	TPM-B2	TPM-B3	TPM-B4	Function annotation	References
B15483	gi|225446176	2.97	0.46	0.01	0.01	zinc finger CONSTANS-like protein	[15]
B3312	gi|170280687	0.01	0.01	1.05	0.01	GIGANTEA (clock-regulated protein)	[36]
B12118	gi|357117348	2.29	2.29	1.88	0.57	MADS-box transcription factor	[37]
B7616	gi|157674589	1.83	0.91	2.72	0.38	FRUITFUL-like protein	[36]
B16198	gi|357124493	10.75	9.15	6.27	1.71	SFT family	[38]
B5152	gi|15232818	0.01	0.46	0.42	0.01	Cycling Dof Factor	[39]
B8880	gi|359474075	0.01	0.69	0.01	0.01	BEL1-like HD transcription factor	[40]
B7173	gi|55741123	626.1	325	198.6	3.98	Sucrose synthase	[11]
B1387	gi|10505183	0.01	0.01	0.01	0.38	Lipoxygenase	[41]
B2632	gi|39777289	1.83	1.41	0.63	0.01	Phytochrome B	[42]

#### qPCR gene expression analysis

**Fig 3:**
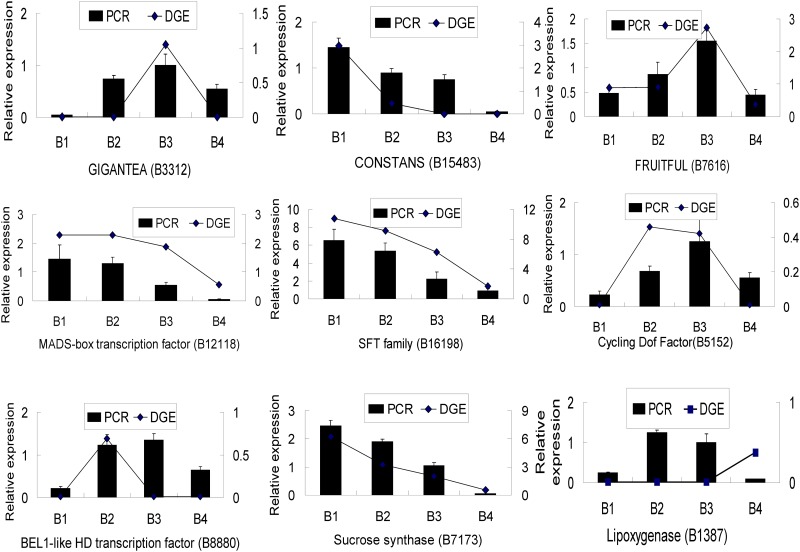
Validation of tag-mapped genes from the four stages of the Chinese water chestnut by qPCR.

To further monitor gene expression profiling, nine genes related to corm formation, including zinc finger protein CONSTANS-LIKE, GIGANTEA-like protein, MADS-box transcription factor, SFT2 transport protein, dof zinc finger protein DOF1.7, sucrose synthase, lipoxygenase, FRUITFUL-like protein, and BEL1-like HD transcription factor, underwent qPCR analysis of transcriptional levels. A similar gene expression pattern was observed for seven of the genes by qPCR and tag sequencing methods during corm formation, suggesting that the results of the two techniques correlate with each other. Only one gene showed a difference in expression by the qPCR technique compared with the tag sequencing analysis (Fig 3).

## Discussion

The high-throughput tag sequencing technique is an effective approach for monitoring gene expression of the whole plant genome, and many metabolic mechanisms have been identified using this method [34,43]. It was previously demonstrated that storage organ formation is regulated by both genetic and environmental factors [15]. In this study, approximately five million tags were identified at the stolon stage, initial stage, middle swelling stage, and later swelling stage (B1, B2, B3, and B4), with 283,469, 245,690, 209,624, and 251,126 distinct tags, respectively. However, only 52.93%, 52.02%, 51.08%, and 40.53% of genes at the four stages were mapped to reference genes because of the unavailability of a complete genome of the Chinese water chestnut (Tables 1 and 2).

COG and GO function classification were applied to analyze these expressed genes, and detailed information is shown in S3 and S4 Figs. Some genes relevant to corm formation were found, and some differences in gene expression between tag sequencing analysis and qPCR were identified. This could be explained by differences in sample collection conditions between the two methods. Alternatively, differences in the corm development stage may have caused differences in expression profiling. Samples were collected according to corm size and classified into the same developmental stage, although differences in growth periods could cause some corms to be larger or smaller than average. Additionally, RNA-seq focuses on the whole transcriptome, so disturbances of bias during sequencing are difficult to avoid, and gene expression changes are determined according to the number of reads. However, qPCR concentrates on the expression of a specific gene so is more reliable at reflecting gene transcription during plant metabolism.

### Survival in an Anaerobic Environment

The entire growth season of the Chinese water chestnut must be maintained in shallow water, so the plant would be expected to develop molecular mechanisms to adapt to anaerobic conditions. We observed that many genes showed an altered expression profile during corm formation (Fig 2), of which some were related to anaerobic adaptation such as genes encoding alcohol dehydrogenase (B4394), NADH dehydrogenase (B2836), superoxide dismutase (B13999), CDPK (B806), MYB transcription factor (B8501), ethylene responsive transcription factor (B490), and ATPase (B227); these showed increased expression during corm formation (Table 3 and S2 Table). These genes also showed enhanced expression in the lotus root and arrowhead during storage organ formation [10, 44].

Many reports show that plant products and quality are often destroyed by anaerobic environments [45,46,47,48], which can lead to the starvation of people who depend on these crops. Compared with these xerophytes, aquatic plants such as the lotus root, arrowhead, water celery, and Chinese water chestnut show increased adaptations to anaerobic conditions [44,49]. The expression of some key genes, including ethylene responsive factor, alcohol dehydrogenase, *SOD*, and *HSP*, is an essential strategy for plants to survive during storage organ formation [50,51,52,53,54]; therefore, increased transcriptional levels of alcohol dehydrogenase are necessary for aquatic plants to maintain normal growth under anaerobic conditions.

### Hormonal Signal Transduction during Corm Formation

Formation of the storage organ is affected by genetic and environmental factors [11], and hormonal signal transduction is necessary for its development. We found that several ethylene-responsive factors (B0/B1: ethylene-responsive transcription factor 9; B2/B3: ethylene-responsive transcription factor 1A, and ethylene-responsive transcription factor CRF4; B3/B4: ethylene-responsive transcription factor 4, ethylene-responsive transcription factor 5-like, and ERF1 transcription factor) showed enhanced expression during corm formation (Tables 3 and 4), suggesting that the ethylene signal transduction pathway is necessary for corm development in the Chinese water chestnut.

Ethylene is involved in various biological processes [55, 56], and has been suggested to be a promoter of plant growth and development or the anaerobic response [57]. Aside from biotic and abiotic stress, ethylene has many other functions, such as cell division, plant height, and the underground formation of storage organs [58]. Constitutive expression of an AP2 domain class transcription factor previously resulted in increased resistance to abiotic stress in the potato plant, and a transgenic plant showed decreased cell size, plant height, hypocotyl elongation, and fertility [59]. It has also been reported that transgenic plants expressing some AP2 domain class transcription factors have altered flowering times [60]. WRI1, a putative AP2 domain class transcription factor, was reported to regulate seed development in *Arabidopsis*, and transgenic plants with this gene have a higher content of seeds and triacylglycerols [61]. The storage content is altered in transgenic rice plants with an AP2 domain class transcription factor because of the control of expression of the waxy gene [62]. In this study, we found that several ethylene-responsive factors and AP2 domain class transcription factors showed enhanced expression during corm formation, and we believe that expression of AP2 domain class transcription factors is relevant to the development of the Chinese water chestnut storage organ.

There is increasing evidence for GA and JA in storage organ formation. Stolon elongation was previously found to be promoted and storage organ formation inhibited by a high GA content [63]. An increased GA content in transgenic plants with the GA oxidase gene requires a longer duration of short-day photoperiods for storage organ formation, while this formation is accelerated when enzyme activity is decreased [17]. In a dwarf mutant of *Solanum tuberosum* ssp. *Andigena*, storage organ formation occurred during both long and short days because the GA content was decreased. Interestingly, it has also been shown that formation of the storage organ can be affected by conditions of short days when GA biosynthesis is totally inhibited [19]. This evidence indicates that GA plays an important role in storage organ formation, which is directly regulated by the photoperiod. In the present study, expression of the GA 20-oxidase gene (B15737), which is involved in GA biosynthesis, was increased during B1/B2 stages (the stolon stage to the initial stage) (Table 3), suggesting that a high level of GA probably benefited elongation of the stolon.

JA has also been shown to be involved in plant metabolism and the stress response [64, 65]. For example, endogenous JA was identified as a necessary inducer of storage organs in potatoes and yams [66]. Ravnikar et al. (1993) suggested that exogenous JA is involved in garlic bulb formation [67], while Zeas et al. (1997) suggested that JA not only induces the formation of storage organs, but also increases their number. The same phenomenon is also found in *Pterostylis sanguinea*, where the exogenous application of JA promotes tuber formation [68].

Some genes, including MYB, transcription factor bHLH, bZIP, AP2/ERF domain-containing transcription factor, and lipoxygenase, which have previously been demonstrated to promote organ formation [69,70,71,72,73], were increased during corm formation in the present study. We list some genes with a role in storage organ formation [74,75,76,77,78] in Table 5. Overall, the expression profiles of these genes show that corm formation is highly complex and regulated by multiple genes.

### DEGs Involved in the Ca^2+^ Signal Transduction Pathway during Corm Formation

We found that three proteins relevant to Ca^2+^ signaling, including a calmodulin-like protein (B18064), CaM-binding transcription factor (B40), and a calcium-dependent protein kinase (B2484), showed enhanced expression during corm formation (Tables 2 and 5). Ca^2+^ is a second messenger molecule that is involved in many biological processes [79]. Calmodulin perceives the signal when Ca^2+^ and CaM are combined (Ca^2+^/CaM), and then modulates the cellular response [80]. Ca^2+^/CaM has also been shown to be closely associated with storage organ formation [81]. In transgenic potato plants, constitutive expression of CaM affects the underground shape and size of tubers [82]. CaM-binding proteins were also reported to be involved in the formation of storage organs [83, 84]. These findings indicate that the Ca^2+^ signal transduction pathway is necessary for the development of storage organs. Therefore, the increased expression of calmodulin-like protein, CaM-binding transcription factor, and calcium-dependent protein kinase genes in our present study further demonstrates that Ca^2+^ signaling is critical for the formation of storage organs.

## Conclusions

We analyzed gene expression during corm formation in the Chinese water chestnut using high-throughput tag sequencing. In total, 4,371,386, 4,372,602, 4,782,494, and 5,276,540 clean tags, including 119,676, 110,701, 100,089, and 101,239 distinct tags, were obtained at each stage, respectively (B1: the stolon stage, B2: the initial swelling stage, B3: the middle swelling stage, and B4: the late swelling stage). Of these, 11,592, 10,949, 10,585, and 7,111 genes were annotated from the B1, B2, B3, and B4 stage, respectively, after mapping their functions in existing databases. A number of DEGs were found in the B1/B2, B2/B3, and B3/B4 stages during corm formation, and 10 important storage organ formation genes were also identified. qPCR results were highly correlated with those of tag sequencing analysis.

## Supporting Information

S1 Fig Developmental stages of the Chinese water chestnut, including the stolon stage, the initial stage, the middle swelling stage, and the later swelling stage. (JPG)

S2 Fig Sequencing saturation analysis of four libraries. B1: stolon stage; B2: initial swelling stage; B3: middle swelling stage; B4: later swelling stage.
(JPG)

S3 Fig GOG function classification of genes expressed during corm formation. All genes identified in the B1/B2, B2/B3, and B3/B4 libraries were classified into 25 classifications according to their function.
(JPG)

S4 Fig GO analysis of genes expressed during corm formation. The percentage and number of genes involved in biological process, cellular component, and molecular function was analyzed using GO Slim Assignment.
(JPG)

S1 Table Information on the genes expressed in the B1/B2, B2/B3, and B3/B4 libraries. (DOC)

S2 Table Primers for genes related to corm formation. (XLS)
