# Supplementary figures and images for: Transcriptome Analysis of Gene Expression during Chinese Water Chestnut Storage Organ Formation
Source: PLoS One. 2016 Oct 7;11(10):e0164223. doi: 10.1371/journal.pone.0164223 (PMC5055346; doi:10.1371/journal.pone.0164223)

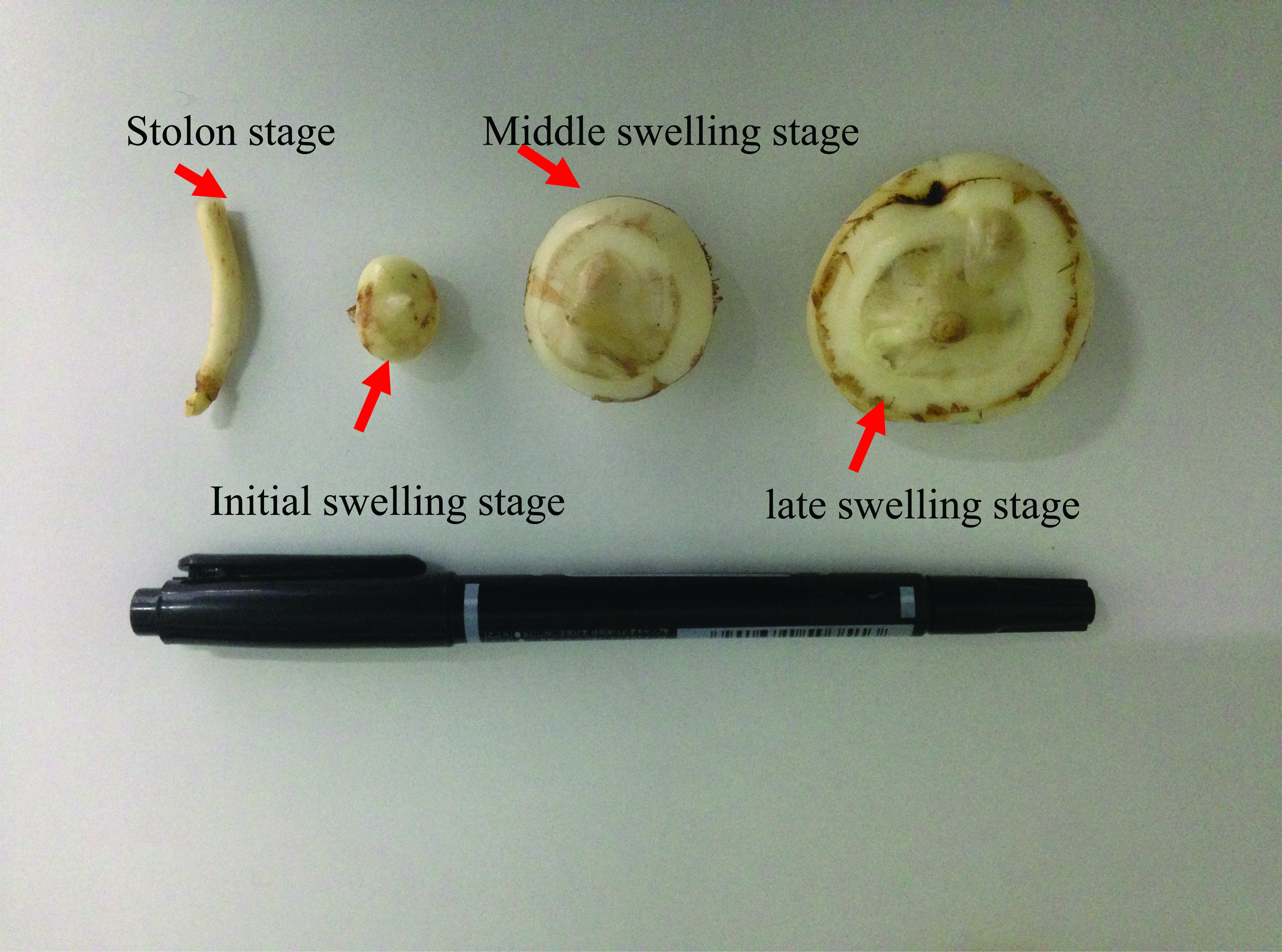

Supplement: S1 Fig — (JPG) [file pone.0164223.s001.jpg]

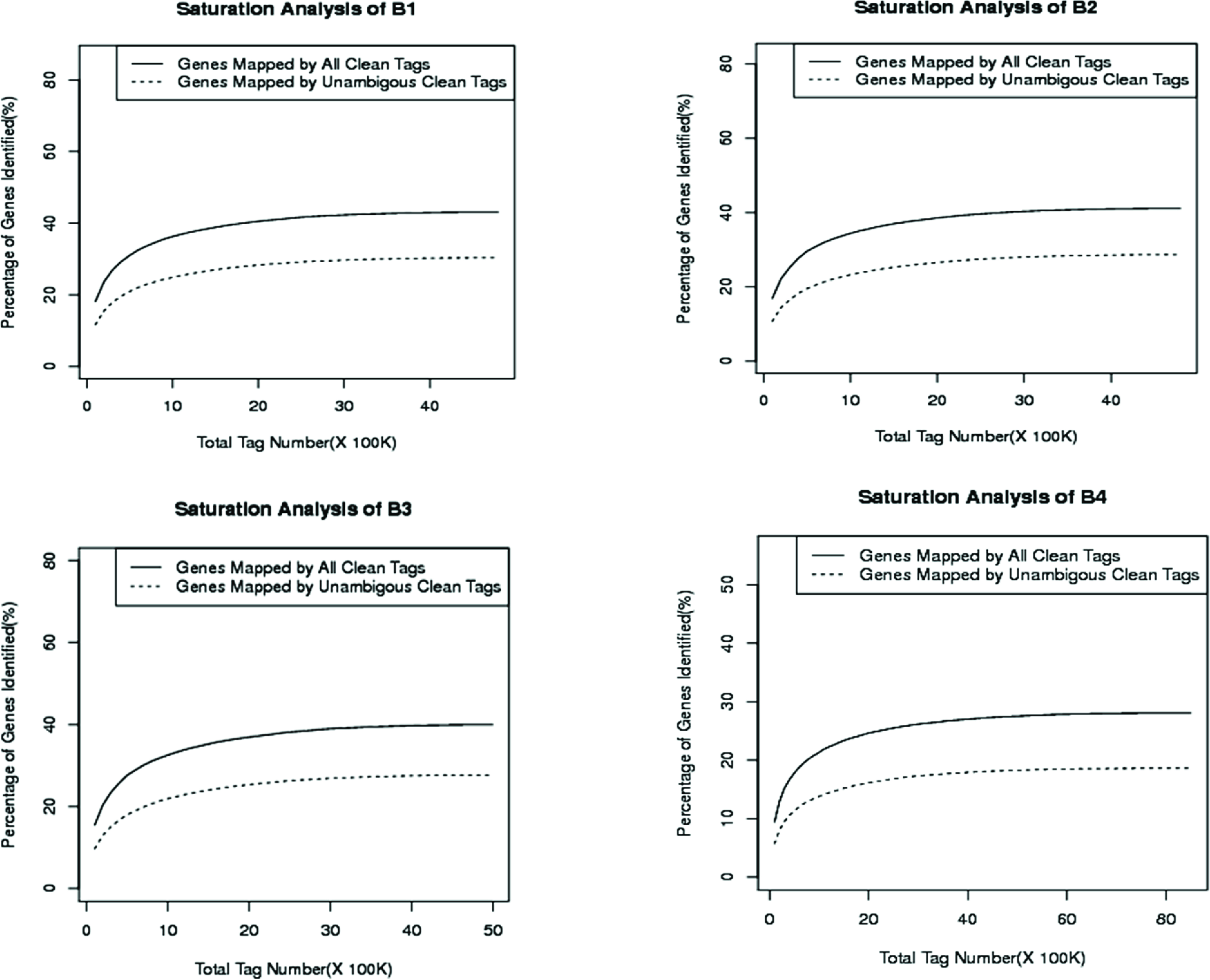

Supplement: S2 Fig — B1: stolon stage; B2: initial swelling stage; B3: middle swelling stage; B4: later swelling stage. (JPG) [file pone.0164223.s002.jpg]

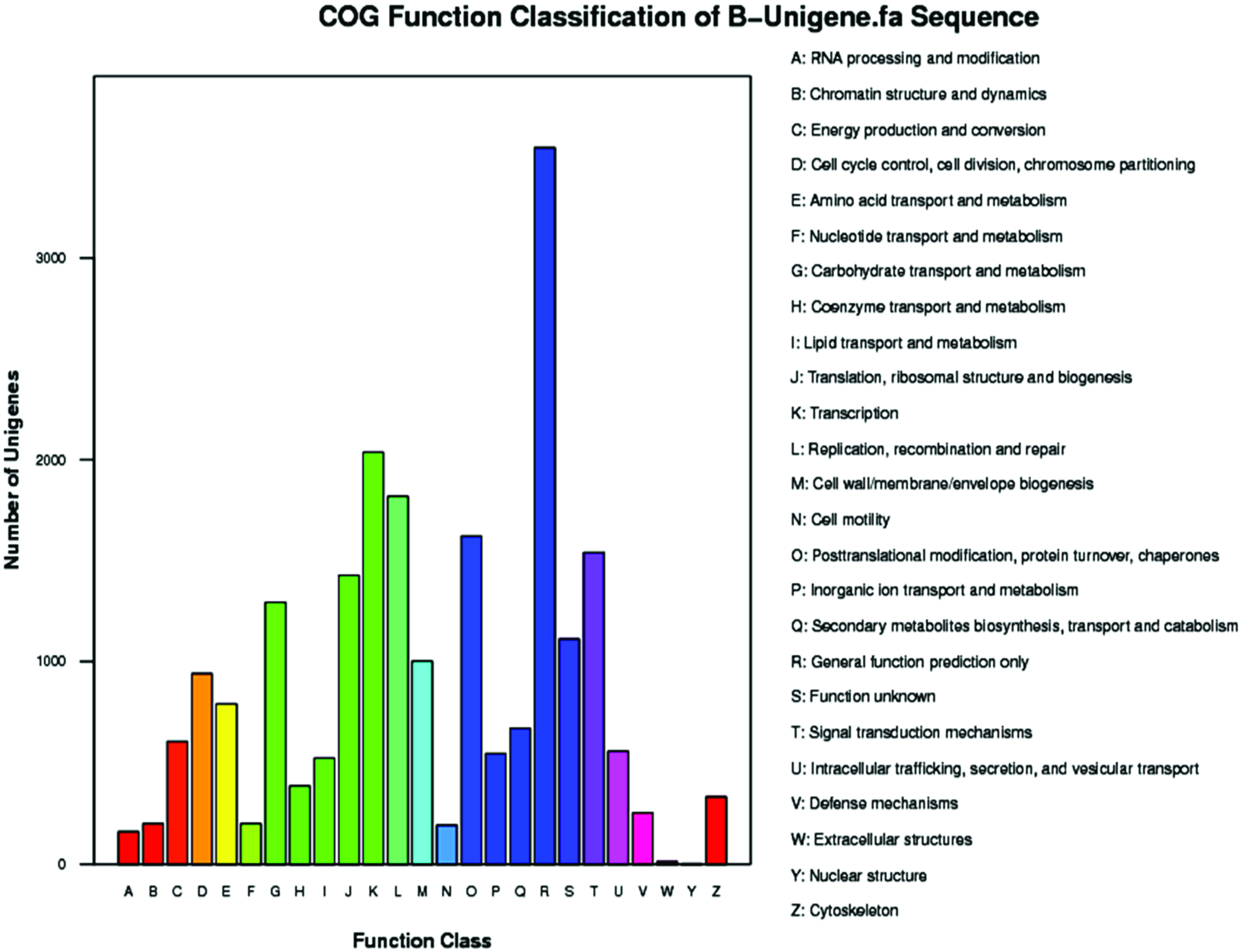

Supplement: S3 Fig — All genes identified in the B1/B2, B2/B3, and B3/B4 libraries were classified into 25 classifications according to their function. (JPG) [file pone.0164223.s003.jpg]

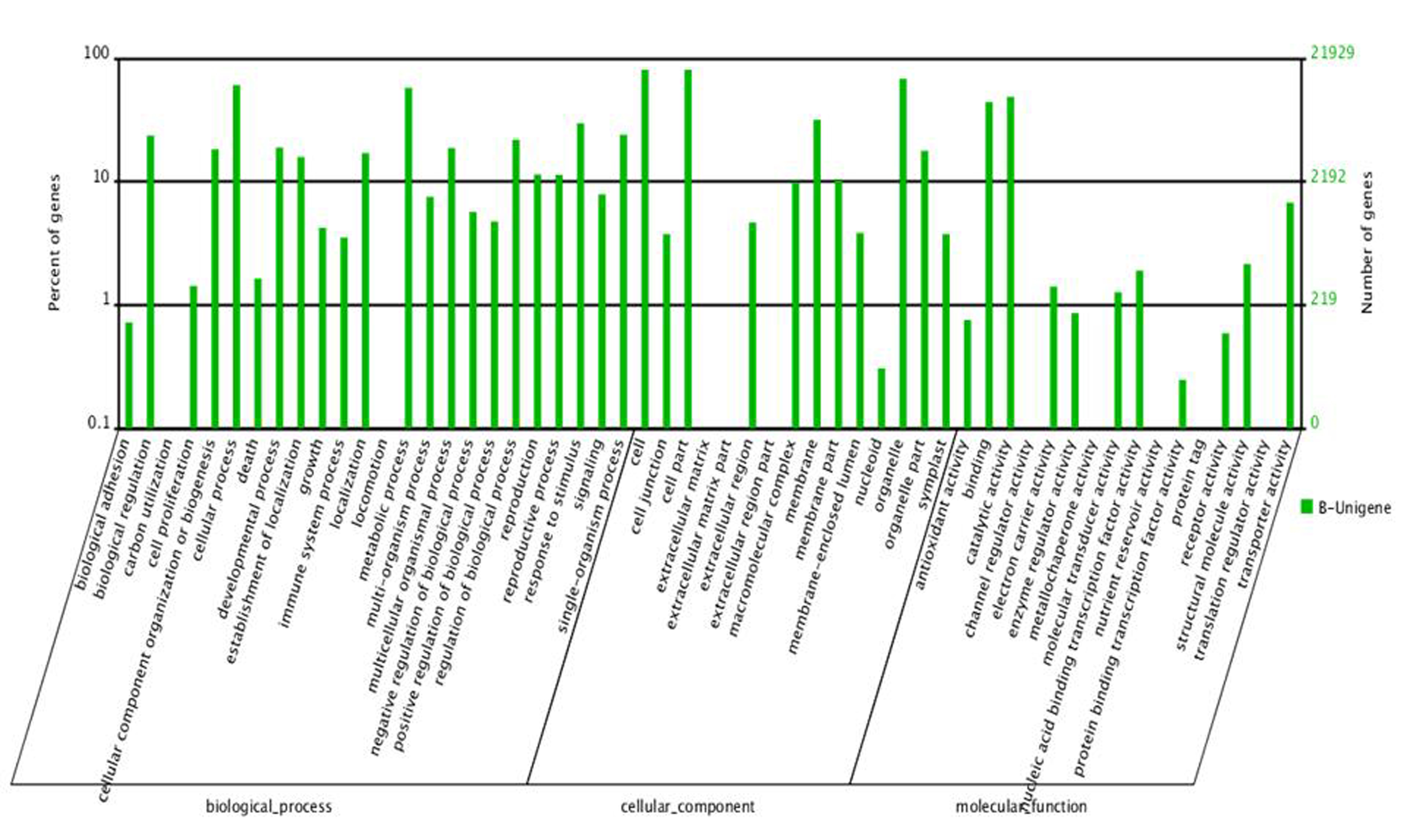

Supplement: S4 Fig — The percentage and number of genes involved in biological process, cellular component, and molecular function was analyzed using GO Slim Assignment. (JPG) [file pone.0164223.s004.jpg]
