# Supplementary material for: Transcriptome Analysis of Gene Expression during Chinese Water Chestnut Storage Organ Formation
Source: PLoS One. 2016 Oct 7;11(10):e0164223. doi: 10.1371/journal.pone.0164223 (PMC5055346; doi:10.1371/journal.pone.0164223)
Supplement: S1 Table — (DOC) [file pone.0164223.s005.doc]

| Gene | Forward primer (5'-3') | Reverse primer (5'-3') | Tm  (^o^C) | Product (bp) |
| --- | --- | --- | --- | --- |
| CONSTANS-LIKE | CTTCACACGCCTTCTTACAT | TGGAGAAAGTAGTGGTGGAG | 59 | 223 |
| GIGANTEA | CCCTTGTCTTTTCACTTTTC | GCCTTCTGTTTGTTGTGTTA | 56 | 170 |
| MADS-box transcription factor | CATATTTAGGGAGGGAAGTG | TGCAGAATTTAGAGCAACAG | 57 | 263 |
| SFT2 | CTGTGAAGCACCATAGAAAC | GCAACTGGAGTGTTCTTCAT | 58 | 242 |
| Dof zinc finger protein | GATTCAAACCGTCTAACAGG | CTACAATCTCTCCCAACCTC | 59 | 311 |
| Sucrose synthase | CATCAGGTATTAAACGGGTC | ATCTCCACACGGTTACTTTG | 58 | 182 |
| Lipoxygenase | GATCACTGTTCCTTTTCTCG | ATCCTGACTACCCTCATTTG | 58 | 379 |
| Phytochrome B | GTTGGTGGGTAAACCGTATA | GCCAGGATATCGAGAAGTTC | 59 | 264 |
| Soluble starch synthase 3 | TTATCCTCCTCTCTTCCTGA | CTGGTGGTATGTTGATGTTG | 58 | 209 |

Table S1. Primers used for qPCR identification
